# Predictors of Multiple Sexual Partners among Men in Ethiopia: A Multilevel Analysis

**DOI:** 10.4314/ejhs.v32i4.4

**Published:** 2022-07

**Authors:** Abebe Debu Liga, Yasin Negash Jabir, Belete Adelo Wobse, Reta Habtamu Bacha

**Affiliations:** 1 Department of Statistics, College of Natural and Computational Sciences, Wolkite University, Ethiopia; 2 Department of Statistics, College of Natural Science, Jimma University, Ethiopia

**Keywords:** multiple sexual partnerships, predictor, multilevel logistic regression, EDHS

## Abstract

**Background:**

Multiple sexual partnerships were one of the public health issues in the spread of high rates of sexually transmitted infections in sub-Saharan regions. An increase in the number of non-marital sexual partners can lead to a loss of satisfaction as well as other mental health repercussions such as greater rates of anxiety, depression, etc. This study examined the predictors of multiple sexual partners among men in Ethiopia.

**Methods:**

This study used 2016 nationally representative data which was conducted using a multistage stratified cluster sampling method. Multilevel binary logistic regression models were employed to estimate the predictors of multiple sexual partners among men in Ethiopia with the assistance of the STATA software.

**Results:**

In this study 6778 participants were considered with an overall prevalence rate of multiple sexual partners of 6.5% during the 12 months preceding the survey. The findings showed that older-age, urban-resident, inconsistent use of a condom, exposure to any media, abuse of alcohol, early-time first-sex, and religion were predictors of multiple sexual partners among men in Ethiopia.

**Conclusions:**

The findings revealed that the prevalence rate of men's multiple sexual partners in Ethiopia was very high. Therefore, the country needs to re-examine the behavioral change strategies periodically to adapt to the contextual realities and engage relevant stakeholders. Specifically, health sectors and religious organizations should develop strategies to create awareness in society on the risk of having multiple sexual partnerships. In addition, we highly recommend stakeholders prepare risk reduction interventions that take the significant predictors of multiple sexual partners.

## Introduction

Multiple sexual partnerships (MSP) were defined as having more than one sexual partner over a specific period and it is extensively believed that one of the global problems that put many people at risk for sexually transmitted infections (1–3). These infections are the common methods of the cause of the high prevalence of risky sexual behaviors such as inconsistent use of a condom, lack of male circumcision, abuse of substances, and multiple sexual relationships (4–8).

In many nations, especially in Sub-Saharan Africa, such as Ethiopia, multiple sexual partnerships are a public health issue (9,10). It increases the risk of contracting sexually transmitted infections (STIs), such as HIV (11). People who have several sexual partners put their monogamous partners at risk of contracting STI (12). Furthermore, an increasing number of sexual partners has been strongly associated with an increased risk of substance use, especially among women (13). In addition, an increase in the number of non-marital sexual partners can lead to a loss of satisfaction as well as other mental health repercussions such as greater rates of anxiety and depression, as well as deterioration in the quality of connections with family, friends, and the community as a whole (14,15).

According to a previous study in sub-Saharan Africa, the prevalence of male MSPs ranges up to 29% (2, 16). A study conducted by Exavery in Tanzania revealed that among 7.8% of sexually active women (17) and Wilson Chialepeh in Malawi found that 69% of males and 35.4% of females (18) had two or more sexual partners before one year preceding the survey. Similarly, a study done by Shayo and Kalomo revealed that the overall prevalence of sex with multiple partners was 20·9% (19).

According to the Ethiopian Demographic and Health Survey 2016, they reported that there were 3% of young sexually active males aged 15–49 years had had multiple sexual partners and 7% of them had sexual intercourse with neither their wives nor lived together in the 12 months before the survey (20). Evidence has revealed that such factors as education, employment status, occupation, age, substance abuse, religion, wealth, media exposure, age at first sexual intercourse, residence, initiating sex at a younger age, partner type, negative attitude towards the status of women, positive attitude towards MSP, and region are associated with MSPs (2, 17–18, 20–33). In addition, studies showed that a spectrum of biological, behavioral, and traditional issues has been examined as the associated factors of multiple sexual partners among young people (18,34) and at regional levels (23).

Even though there were numerous researches conducted on the impact of HIV/AIDS in Ethiopia including age-disparate, age of sexual debut, and the use of a condom, there have been limited studies on the influence of risky sexual behaviors like multiple sexual partnerships (MSPs). In Ethiopia, there is a high incidence of sexual issues and nowadays increases multiple sexual practices somewhat. All of these findings present the risks and consequences of multiple sexual partners using a variety of statistical methods, and few studies were done on multiple sexual partners in Ethiopia. With these factors in mind, this study assessed the predictors of multiple sexual partners among men in Ethiopia using a nationally representative survey based on multilevel binary logistic regression analysis. The purpose of implementing a multilevel binary logistic regression model was to handle random effects, regional variation, and subject-specific variation of multiple sexual partners (MSPs). Therefore, this study intended to identify the cause of factors that explains the variation among men from multiple sexual partners between and within regions and, to determine the prevalence of multiple sexual partners in Ethiopia using multilevel analysis.

## Methods

Data source and sampling procedure: This study was based on the 2016 Ethiopian Demographic and Health Survey data, which was the fourth survey at the national level and conducted by the Central Statistical Agency from January 18 to June 27, 2016. The survey included all regional states of Ethiopia namely, Tigray, Afar, Amhara, Oromia, Somali, Benishangul, Southern Nations Nationalities and People Region, Gambela, and Harari) and two administrative cities, Addis Ababa and Dire Dawa. The data was conducted using a community-based multistage stratified cluster sampling study method. All men aged 15–59 years were included in the interview (20). The survey collected information on a total of 14795 men with an accomplished respondents of 12,688 men were interviewed face to face on their background characteristics as well as different health issues, out of which only 6778 men were completed measurements on multiple partnership parameters, and hence we considered it in this study.

**Response ariable**: Multiple sexual partners were defined as having two or more sexual partners during the 12 months before the survey. It was coded as “1” when the participant had two or more sexual partners and “0” when there was one sexual partner.

**Predictor variables**: Based on the reviewed literature, the common predictors that are expected to influence multiple sexual partners were age, residence, region, educational status, wealth index, age at first sex, working status, religion, media exposure, smoking habit, use of a condom, chawing Khat, use of alcohol, and ever tested for HIV.

**Inclusion and exclusion criteria**: All males having multiple sexual partners were included and those who do not have were excluded from the study.

**Methods of data analysis**: Descriptive statistics such as frequencies and percentages were performed to summarize the categories of the factor and, a chi-square test was done to illustrate the statistically significant association between predictor variables, and multiple sexual partners.

While multilevel binary logistic regression models were conducted to examine the influence and variations of multiple sexual partners across the regional state of Ethiopia with the assistance of the STATA version 16 software packages. This model is appropriate for studies having nested data with organizing more than one level (36). The units of analysis are usually individuals at a lower level nested within integrated units at a higher level. A multilevel binary logistic regression model is an extension of a single-level logistic regression model by including random effects from the model (36). In this study, a two-level logistic regression model was taken into account by men considered as level 1 and region considered as level 2 to comprehend unexplained variation within groups and the unexplained variation between groups as random variability. Because not only unexplained variation in multiple sexual partners between men, it also unexplained variation between regions is regarded as a random variable. In this study, three multilevel logistic regression models were fitted hierarchically namely; model-1 or null model (model without predictors), or no explanatory variable was included. This model represented the total variance in the predictor of multiple sexual partners among regions. In model-2, only individual-level factors were included. Model-3 assessed the effects of individual-level and regional-level factors.

Hosmer and Lemeshow Test of Goodness of fit: The Hosmer and Lemeshow Goodness of fit test is the most commonly used to measure the goodness of fit for categorical data. A goodness of fit test tells how well the data fits the model (37).

**Test of heterogeneity between regions**: To test whether there are systematic differences between the groups (regions); the familiar chi-square test for the contingency table was used (38).

**Model comparison and ICC**: The deviance, AIC, and BIC values of the random intercept multilevel logistic model were the minimum among the deviance, AIC, and BIC values of the null and random coefficient models indicating that it was the most effective model to predict the occurrence of multiple sexual partners. Furthermore, the intraclass correlation coefficient (ICC) measures the proportion of variance in the outcome explained by the grouping structure.

**Ethics approval and consent to participate**: It is not necessary due to the use of previously published data by the CSA of Ethiopia.

## Results

**Results of descriptive statistics**: A total of 6778 participants were included in the study. Among these, 439 (6.5%) of them had two or more sexual partners during the 12 months preceding the survey. Table 1 presents the distribution of the respondents by different variables in the study. It showed that the mean, median, minimum, and maximum age of the participants was 37.31, 36, 16, and 59, respectively. Among the total participants, 7.1%, 34.7%, 33.1%, and 25.1% were between the age of 15–24 years, 25–34 years, 35–44 years, and ≥45 years respectively facing the highest rate (10.7%) of multiple sexual partners aged ≥45 years relative to lowest rate (1.9%) were verified in aged 15–24 years. Out of the total, 24.3% of the respondents were from the urban part with experienced the highest rate of multiple sexual partners (7.7%). *Table 1* also shows that among the total participants facing two or more sexual partners, 0.0%, 0.1%, 15.3%, 0.7%, 8.5%, 14.5%, 10.3%, 8.5%, 9.1%, 4.0%, and 2.6% of them lived in Addis Ababa, Tigray, Afar, Amhara, Oromia, Somali, Benshangul, SNNPR, Gambella, Harari, and Dire-Dawa regions in Ethiopia respectively.

**Table 1:** Distribution of multiple sexual partners by selected variables

Variables	Category	Counts (%)	Multiple Sexual Partner		P-value

			Had one partner	Had two or more partner	
	15–24 Years	480(7.1)	98.1%	1.9%	
Age group	25–34 Years	2354(34.7)	96.2%	3.8%	<0.0001
	35–44 Years	2241(33.1)	92.9%	7.1%	
	≥45 Year	1703(25.1)	89.3%	10.7%	
Place of Residence	Urban	1647(24.3)	92.3%	7.7%	<0.0001
	Rural	5131(75.7)	97.4%	2.6%	
	Addis Ababa	476(7)	100%	0.0%	
	Tigray	668(9.9)	99.9%	0.1%	
	Afar	398(5.9)	84.7%	15.3%	
	Amahara	957(14.1)	99.3%	0.7%	
	Oromia	973(14.4)	91.5%	8.5%	
Region	Somali	592(8.7)	85.5%	14.5%	<0.0001
	Benishangul	584(8.6)	89.7%	10.3%	
	SNNP	904(13.3)	91.5%	8.5%	
	Gambela	427(6.3)	90.9%	9.1%	
	Harari	379(5.6)	96.0%	4.0%	
	Dire Dawa	420(6.2)	97.4%	2.6%	
Educational	None	2682(39.6)	91.9%	8.1%	
	Primary	2571(37.9)	93.0%	7.0%	<0.0001
Level	Secondary	770(11.4)	97.0%	3.0%	
	Higher	755(11.1)	97.4%	2.6%	
	Orthodox	2746(40.5)	98.0%	2.0%	
Religion	Muslim	2778(41.0)	90.2%	9.8%	<0.0001
	Protestant	1148(16.9)	91.4%	8.6%	
	Other	106(1.6)	87.7%	12.3%	
	High	2907(42.9)	96.1%	3.9%	
Wealth index	Middle	999(14.7)	93.2%	6.8%	<0.0001
	Low	2872(42.4)	91.0%	9.0%	
Smoke habit	No	5968(88.0)	94.0%	6.0%	<0.0001
	Yes	810(12.0)	90.2%	9.8%	
Ever Used	No	6602(97.4)	93.4%	6.6%	0.009*
Condom	Yes	176(2.6)	98.3%	1.7%	
Currently	No	386(5.7)	90.9%	9.1%	0.033*
Working	Yes	6392(94.3)	93.7%	6.3%	
Ever been	No	3136(46.3)	91.5%	8.5%	<0.0001
Tested HIV	Yes	3642(53.7)	95.2%	4.8%	
Ever Chewed	No	4234(62.5)	93.8%	6.2%	>0.05
Khat	Yes	2544(37.5)	93.0%	7.0%	
Ever Drink	No	3610(53.3)	92.2%	7.8%	<0.0001
Alcohol	Yes	3168(46.7)	95.0%	5.0%	
Any Media	No	2270(33.5)	94.4%	5.6%	0.028*
Exposure	Yes	4508(66.5)	93.1%	6.9%	
Age at First	≤15 Year	502(7.4)	89.6%	10.4%	<0.0001
Sex	16–19 Year	2244(33.1)	93.1%	6.9%	
	≥20 Year	4032(59.5)	94.2%	5.8%	

Most (42.9%) had a high wealth index with experiencing the lowest rate of multiple sexual partners (3.9%). Similarly, 39.6% who had no education faced the highest rate of multiple sexual partners (8.1%). Among the total, 40.5%, 41%, 16.9%, and 1.6% of the participants followed Orthodox, Muslim, Protestant, and Other religions, respectively. The highest rate of multiple sexual partners had recorded in other religions (12.3%) followed by Muslim (9.8%) and Protestant (8.6%) religion compared with the lowest rate was verified in Orthodox religion (2%).

Furthermore, a summary of the results from the *table* shows that 12% of the participants had no history of smoking and the majority of respondents, 97.4% have not used a condom with a high rate of multiple sexual partners (6.6%). Likewise, out of the total, 94.3% of the participants had work and 62.5% of them had a history of chewing Khat. A majority (55.65%) had their first sexual intercourse aged ≥20 years, and 66.5% of them used media. In addition, more than half (53.7%) had ever drunk alcohol and had been tested for HIV (53.7%).

The Chi-square test of association in Table 1 showed that age, residence, region, education, religion, wealth index, smoking habit, ever used condom, currently working, ever been tested for HIV, ever drunk alcohol, media exposure, and age at first sex had a significant association with multiple sexual partners at 5% level of significance. A summary result presented in *Table 2* shows that the p-value of the test statistic is greater than 0.05, which pointed out the fitted model is good for the study dataset.

**Table 2:** Hosmer and Lemeshow Test

Hosmer and Lemeshow Test			
Step	Chi-square	Df	Sig.
1	10.30	8	0.245

**Results of Multilevel Binary Logistic Regression Model**: Table 3 indicated that the Chi-square value of 292.329 with p ≤ 0.001, which is significant and shows that there is heterogeneity among multiple sexual partners across regional states of Ethiopia. Therefore, we can apply multilevel logistic analysis. Predictors with a p-value less than 0.05 were selected, which had a significant association with multiple sexual partners in the final model. The most varying predictor over the region compared with other variables was age and which was permitted to analyze the random slope model.

**Table 3:** Tests of Heterogeneity

Chi-Square Tests			
Statistics	Value	Df	p-value
Pearson Chi-Square	292.329	10	<0.001

The deviance, AIC, and BIC values were employed to select the best model among the three fitted multilevel models in Table 4. The ICC in the null model from *table 4* was 0.5097, showing that 50.97% of the variation in the prevalence of multiple sexual partners can be explained by grouping in regions ((higher-level units). The remaining 49.03% of the variation is explained within the region (lower level units).

**Table 4:** Summary results of model selection criteria and ICC

Class	Null model	Random intercept model	Random coefficient model
Deviance-based χ^2^ (p-value)	309.94 (<0.001)	236.9632 (<0.001)	239.5636 (<0.001)
AIC	2946.02	2749.057	2750.456
BIC	2959.663	2899.128	2914.171
ICC	0.5091748	0.425732	0.2993711

**Multilevel Binary logistic regression results of predictors associated with multiple sexual partners**: The summary of multilevel binary logistic regressions shown in Table 5 reveals that age, residence, religion, ever used condom, drinking status of alcohol, media exposure, and age at first sex were significantly associated with the determinants of multiple sexual partners among men in Ethiopia (p<0.05). The log odds of men aged 25–34 years, 35–44 years, and ≥45 years were 2.72 (95% CI: 1.35, 5.49), 5.92 (95% CI: 2.95, 11.85), and 10.47 (95% CI: 5.21, 21.04) times more likely to experience multiple sexual partners than 15–24 years age respectively. Men's living in the urban part had 1.61 (CI: 1.08, 2.39) times more likely to have multiple sexual partners than in the rural part. Male's following Muslim, Protestant, and Other religions had 2.04 (95% CI: 1.43, 2.91), 1.85 (95% CI: 1.26, 2.71), and 2.29 (95% CI: 1.15, 4.53) times higher odds of experiencing multiple sexual partners than Orthodox followers. The log odds of men's ever used condoms were 0.24 (95% CI: 0.07, 0.76) times less likely to have multiple sexual partners than those with not ever used condoms. The log odds of men who had not drunk alcohol were 0.60 (95% CI: 0.49, 0.75) times less likely to experience multiple sexual partners than those who had ever used alcohol.

**Table 5:** Multilevel Binary logistic regression results of predictors associated with multiple sexual partners

Variable	Category	Model 1 Odds Ratio (95% CI)	Model 2 Odds Ratio (95% CI)	Model 3 Odds Ratio (95% CI)
Age (in year)	15–24 (Ref)	___		
	25–34	___	2.72(1.35, 5.49)	2.51(1.17, 5.39)
	35–44	___	5.92(2.95, 11.85)	4.77(1.94, 11.75)
	≥45	___	10.47(5.21, 21.04)	7.33(2.44, 22.05)
Residence	Rural (Ref)	___		
	Urban	___	1.61(1.08, 2.39)	1.60(1.08, 2.38)
Education	None (Ref)	___		
	Primary	___	1.16(0.91, 1.49)	1.16(.91, 1.48)
	Secondary	___	0.65(0.40, 1.06)	0.64(0.39, 1.04)
	Higher	___	0.70(0.41, 1.20)	0.68(0.40, 1.17)
Religion	Orthodox (Ref)	___		
	Muslim	___	2.04(1.43, 2.91)	2.03(1.42, 2.90)
	Protestant	___	1.85(1.26, 2.71)	1.85(1.26, 2.71)
	Other	___	2.29(1.15, 4.53)	2.31(1.16, 4.58)
Wealth index	High (Ref)	___		
	Middle	___	1.25(0.88, 1.76)	1.24(0.88, 1.75)
	Low	___	1.29(0.96, 1.73)	1.29(0.96, 1.73)
Smoking habit	No (Ref)	___		
	Yes	___	0.97(0.73,1.28)	0.97(0.74, 1.28)
Ever Used	No (Ref)	___		
Condom	Yes	___	0.24(0.07, 0.76)	0.24(0.07, 0.76)
Currently	No (Ref)	___		
Working	Yes	___	1.06(0.70, 1.60)	1.06(.70, 1.60)
Ever been	No (Ref)	___		
Tested HIV	Yes	___	1.17(0.92, 1.47)	1.17(0.92, 1.48)
Ever Drink	Yes (Ref)	___		
Alcohol	No	___	0.60(0.49, 0.75)	0.61(0.49, 0.75)
Any Media	No (Ref)	___		
Exposure	Yes	___	1.39(1.11, 1.74)	1.39(1.11, 1.74)
Age at First	≤15 (Ref)	___		
Sex (in year)	16–19	___	0.90(0.63, 1.28)	0.90(0.63, 1.28)
	≥20	___	0.67(0.47, 0.94)	0.66(.47,0.94)
Constant		0.03(.010, 0.100)	0.003(.0007, 0.010)	0.004(.001, .013)

Moreover, being exposed to any media had 1.39 (95% CI: 1.11, 1.74) times higher odds of experiencing multiple sexual partners compared to non-exposed to any media. Men aged ≥20 years at their first sex had 0.67 (95% CI: 0.47, 0.94) times fewer odds of experiencing multiple sexual partners compared to those with their first sex at age ≤15 years.

## Discussion

This study identified the predictors of multiple sexual partnerships among men in Ethiopia. The finding showed that out of 6778 participants, 6.5% of them had multiple sexual partners during the 12 months preceding the survey. The summary statistics also showed that men who lived in Afar, Somali, Benshangul, and Gambella regions were more likely to experience multiple sexual partners than men who lived in Addis Ababa, Tigray, and Amhara regions. This regional difference is supported by a study conducted by Odimegwu et al., 2019 (39) revealing that regional differences in behavior happen due to socio-cultural practices. This result is also in agreement with a study done by Ojikutu et al., 2016 showing regional issues had been the cause of multiple sexual partners at regional levels of the country (23).

The findings revealed that age, residence, religion, ever used condoms, drinking status of alcohol, media exposure, and age at first sex were significantly associated with the determinants of multiple sexual partners among men in Ethiopia. This study found that older age had higher odds of experiencing multiple sexual partners. This finding is consistent with a study conducted by (26) which showed that older men increased the likelihood of having multiple sexual partners, and contradicted a study done by (18, 24, 25) revealed that being younger increased the odds of having multiple sexual partners. This result may be leading to the greater need to increase the number of families in their household.

The study also revealed that men's living in the urban part had higher odds of experiencing multiple sexual partners than in the rural part. This finding is in agreement with a study conducted by (18, 25–27) which showed that urban residents had more likely to have multiple sexual partners. Men following Muslim, Protestant and other religious categories had more than one sexual partner. This result is in line with a study done by (28) which showed that having more than one sexual partner was accepted by Islamic regions of Machinga and the neighboring districts. And, it is also supported by a study done by (17, 18) who stated as being Muslims were more likely to have multiple sexual partners. This study indicated that men's ever used condoms were less likely to have multiple sexual partners. The finding is consistent with a study conducted by (18, 28) which revealed that nonuse of contraception increased multiple sex partners. Thus, this predictor may have countless roles to reduce the spread of sexually transmitted infections, especially HIV.

The finding revealed that men who drank alcohol have more likely to be exposed to multiple sex partners. This outcome is consistent with previous research findings conducted by (18, 29–32) showed that being abused with alcohol increased multiple sexual partners. This may be leading to alcohol drinking increasing the risk of multiple sex partners due to less prospective to use condoms consistently.

Additionally, the finding showed that being exposed to any media had higher odds of experiencing multiple sexual partners. This result is in line with a study employed by (18) which showed that youths exposed to media at least once a week are more likely to have multiple sexual partners. Similarly, a study conducted by (33) being addicted to internet use is a predictor of the likelihood of having multiple sexual partners, and a study employed by (39) showed that being exposed to mass media was associated with lower odds of having single sexual partners. This is due to mass media increasing the accessibility of people to learn and understand sexual behavior even if it has an important role to distinguish the good and bad side of sexual topics.

Men aged ≥20 years at their first sex had fewer odds of experiencing multiple sexual partners compared to those with their first sex aged ≤15 years. This finding is consistent with a study done by (2, 20, 25) which showed that early time first sex increases the likelihood of multiple sex partners. Early time first sexual intercourse increases the risk of sexually transmitted infections like HIV/AIDS and others.

In conclusion, in this study, 6778 participants were considered with an overall prevalence rate of multiple sexual partners of 6.5% during the 12 months preceding the survey. Based on the findings, we can conclude that the prevalence rate of men's multiple sexual partners in-country was very high. Therefore, the country needs to reexamine the behavioral change strategies periodically to adapt to the contextual realities and engage relevant stakeholders. Specifically, health sectors and religious organizations should develop strategies to create awareness in society on the risk of having multiple sexual partnerships. In addition, we highly recommend stakeholders prepare risk reduction interventions that take into account the significant predictors of multiple sexual partners.

The strength of this study are the representativeness and the large sample size, applying multilevel binary logistic regression models, using a chi-square test of association to handle the heterogeneity, and an appropriate sampling design was applied to collect the study data. While, the limitation of this study are not taking some significant predictors which may influence the outcome variable and other predictors which were not included in the analysis due to missing values and high rates of non-responses such as the number of moves, negative attitude towards the status of women and positive attitude towards MSPs, etc. Thus limitation are lead to handling secondary data for the study.
